# Assessment of nodal staging and risk factors for nodal involvement in gallbladder cancer

**DOI:** 10.1093/bjsopen/zraf056

**Published:** 2025-05-23

**Authors:** Anita Balakrishnan, Petros Barmpounakis, Nikolaos Demiris, Bodil Andersson, Alejandro Brañes, Xavier de Aretxabala, Malin Sternby Eilard, Paul Gibbs, Simon J F Harper, Emmanuel L Huguet, Asif Jah, Vasilis Kosmoliaptsis, Javier Lendoire, Siong S Liau, Shishir Maithel, Jack L Martin, Colin Noel, Raaj K Praseedom, Alejandro Serrablo, Volkan Adsay, the OMEGA Study Investigators, Tomoyuki Abe, Moh'd Abu Hilal, Maria del Mar Achalandabaso Boira, Mustapha Adham, Mohamed Adam, Maryam Ahmad, Bilal Al-Sarireh, Maite Albiol, Nassir Alhaboob, Adnan Alseidi, Houssem Ammar, Akshay Anand, Bodil Andersson, Pantelis Antonakis, Veronica Araya, Stanley W Ashley, Georgi Atanasov, Fabio Ausania, Ricardo Balestri, Abhirup Banerjee, Sudeep Banerjee, Simon Banting, Giedrius Barauskas, Fabian Bartsch, Andrea Belli, Simona Beretta, Frederik Berrevoet, Ramesh Singh Bhandari, Gerardo Blanco Fernandez, Louisa Bolm, Mathieu Bonal, Emre Bozkurt, Andries E Braat, Luke Bradshaw, Konstantinos Bramis, Alejandro Branes, Lyle Burdine, Matthew Byrne, Maria Caceres, Maria Jesus Castro Santiago, Benjamin Chan, Lynn Chong, Ahmet Çoker, Maria Conde Rodriguez, Daniel Croagh, Alyn Crutchley, Carmen Cutolo, Mathieu D'Hondt, Daniel D'Souza, Freek Daams, Raffaele Dalla Valle, José Davide, Mario de Bellis, Marieke de Boer, Celine de Meyere, Philip de Reuver, Matthew Dixon, Panagiotis Dorovinis, Gabriela Echeverría Bauer, Maria Eduarda, Hasan Eker, Joris Erdmann, Mert Erkan, Evangelos Felekouras, Emanuele Felli, Eduardo Fernandes, Eduardo Figueroa Rivera, Andras Fulop, Daniel Galun, Michael Gerhards, Poya Ghorbani, Fabio Giannone, Luis Gil, Emmanouil Giorgakis, Mario Giuffrida, Felice Giuliante, Ioannis Gkekas, Miguel Gomez Bravo, Bas Groot Koerkamp, Oscar Guevara, Alfredo Guglielmi, Aiste Gulla, Rahul Gupta, Amit Gupta, Marta Gutiérrez, Abu Bakar Hafeez Bhatti, Jeroen Hagendoorn, Zain Hajee, Abdul Rahman Hakeem, Hytham Hamid, Sayed Hassen, Stefan Heinrich, Roberto Hernandez-Alejandro, Ryota Higuchi, Daniel Hoffman, David Holroyd, Daniel Hughes, Arpad Ivanecz, Satheesh Iype, Isabel Jaen Torrejimeno, Shantanu Joglekar, Robert Jones, Klaus Kaczirek, Harsh Kanhere, Ambareen Kausar, Zhanyi Kee, Jessica Keilson, Jorg Kleef, Johannes Klose, Brett Knowles, Jun Kit Koong, Nagappan Kumar, Supreeth Kunnuru, Paleswan Joshi Lakhey, Andrea Laurenzi, Yeong Sing Lee, Felipe Leon, Voon Meng Leow, Jean-Baptiste Lequeu, Mickael Lesurtel, Elisabeth Lo, Stefan Löb, Elizabeth Lockie, Peter Lodge, Dolores López Garnica, Victor Lopez Lopez, Linda Lundgren, Nikolaos Machairas, Dhiresh Maharjan, Deep Malde, Marc Mankarious, Guillaume Martel, Julie Martin, Michele Mazzola, Arianeb Mehrabi, Ricardo Memeo, Flavio Milana, George Molina, Leah Monette, Haluk Morgul, Dimitrios Moris, Antonios Morsi-Yeroyannis, Nicholas Mowbray, Francesk Mulita, Edoardo Maria Muttillo, Malith Nandasena, Pueya Rashid Nashidengo, Arash Nickkholgh, Colin Byron Noel, Masayuki Ohtsuka, Arturs Ozolins, Sanjay Pandanaboyana, Nikolaos Pararas, Alessandro Parente, June Peng, Arkaitz Perfecto Valero, Julie Perinel, Konstatinos Perivoliotis, Teresa Perra, Patrick Pessaux, Natalie Petruch, Gaetano Piccolo, Laszlo Piros, Alberto Porcu, Viswakumar Prabakaran, Raj Prasad, Mikel Prieto Calvo, Florian Primavesi, Eva Maria Pueyo Periz, Alberto Quaglia, Jose M Ramia Angel, Ashwin Rammohan, Francesco Razionale, Ricardo Robles Campos, Manas Roy, Sophie Rozwadowski, Luis Ruffolo, Natalia Ruiz, Andrea Ruzzenante, Lily Saadat, Mohamed Amine Said, Edoardo Saladino, Gabriel Saliba, Per Sandstrom, Carlo Alberto Schena, Anthony Scholer, Christoph Schwarz, Lorenzo Serafini, Leyre Serrablo, Pablo E Serrano, Deepak Sharma, Aali Sheen, Vishwanath Siddagangaiah, Michael Silva, Saurabh Singh, Ajith Siriwardena, Michal Skalski, Mante Smig, Faris Soliman, Abhinav Arun Sonkar, Donzília Sousa Silva, Ernesto Sparrelid, Harry V M Spiers, Parthi Srinivasan, Malin Sternby Eilard, Oliver Strobel, Urban Stupan, Miguel Angel Suarez-Munoz, Manisekar Subramaniam, Teiichi Sugiura, Robert Sutcliffe, Hilko Swank, Shibojit Talukder, Lillian Taylor, Prabin Bikram Thapa, Catherine Teh, Asara Thepbunchonchai, Caman Thieu, Navneet Tiwari, Guido Torzilli, Chutwichai Tovikkai, Blaz Trotovsek, Savvas Tsaramanidis, Georgios Tsoulfas, Katsuhiko Uesaka, Garzali Umar, Lucio Urbani, Michail Vailas, Ronald van Dam, Peter van de Boezem, Stijn van Laarhoven, Tomas Vanagas, Mike Van Dooren, Manon Viennet, Luca Vigano, Aarathi Vijayashanker, Celia Villodre, Toshifumi Wakai, Aklile Workneh, Li Xu, Masakazu Yamamoto, Zhiying Yang, Robert Young, Marko Zivanovic

**Affiliations:** Department of Hepatopancreatobiliary Surgery, Cambridge University Hospitals NHS Foundation Trust, University of Cambridge, Cambridge, UK; Department of Surgery, University of Cambridge, Cambridge, UK; Cambridge Clinical Trials Unit—Cancer Theme, Cambridge University Hospitals NHS Foundation Trust, Cambridge, UK; Department of Statistics, Athens University of Economics and Business, Athens, Greece; Cambridge Clinical Trials Unit—Cancer Theme, Cambridge University Hospitals NHS Foundation Trust, Cambridge, UK; Department of Statistics, Athens University of Economics and Business, Athens, Greece; Department of Surgery, Lund University, Skane University Hospital, Lund, Sweden; Department of Hepatopancreatobiliary Surgery, Hospital Sotero del Rio, Puente Alto, Chile; Department of Digestive Surgery, Hepato-Pancreato-Biliary Surgery Unit, Surgery Service, Gallbladder Consortium Chile, Sotero del Rio Hospital and Clinica Alemana, Santiago, Chile; Transplantation Centre, Sahlgrenska University Hospital, and Department of Surgery, Institute of Clinical Sciences, Sahlgrenska Academy, University of Gothenburg, Gothenburg, Sweden; Department of Hepatopancreatobiliary Surgery, Cambridge University Hospitals NHS Foundation Trust, University of Cambridge, Cambridge, UK; Department of Surgery, University of Cambridge, Cambridge, UK; Department of Hepatopancreatobiliary Surgery, Cambridge University Hospitals NHS Foundation Trust, University of Cambridge, Cambridge, UK; Department of Surgery, University of Cambridge, Cambridge, UK; Department of Hepatopancreatobiliary Surgery, Cambridge University Hospitals NHS Foundation Trust, University of Cambridge, Cambridge, UK; Department of Surgery, University of Cambridge, Cambridge, UK; Department of Hepatopancreatobiliary Surgery, Cambridge University Hospitals NHS Foundation Trust, University of Cambridge, Cambridge, UK; Department of Surgery, University of Cambridge, Cambridge, UK; Department of Hepatopancreatobiliary Surgery, Cambridge University Hospitals NHS Foundation Trust, University of Cambridge, Cambridge, UK; Department of Surgery, University of Cambridge, Cambridge, UK; Department of Surgery, University of Buenos Aires, Hospital Dr Cosme Argerich, Buenos Aires, Argentina; Department of Hepatopancreatobiliary Surgery, Cambridge University Hospitals NHS Foundation Trust, University of Cambridge, Cambridge, UK; Department of Surgery, University of Cambridge, Cambridge, UK; Division of Surgical Oncology, Lurie Comprehensive Cancer Centre, Northwestern University, Chicago, USA; Department of Hepatopancreatobiliary Surgery, Cambridge University Hospitals NHS Foundation Trust, University of Cambridge, Cambridge, UK; Department of Surgery, University of Cambridge, Cambridge, UK; Gastrointestinal Surgery and Hepatopancreatobiliary Surgery, Department of Surgery, University of the Free State, Bloemfontein, South Africa; Department of Hepatopancreatobiliary Surgery, Cambridge University Hospitals NHS Foundation Trust, University of Cambridge, Cambridge, UK; Department of Surgery, University of Cambridge, Cambridge, UK; Department of Hepatopancreatobiliary Surgery, Miguel Servet University Hospital, Zaragoza, Spain; Department of Pathology, Koç University Hospital, Istanbul, Turkey; Department of Pathology, Koç University Research Centre for Translational Medicine (KUTTAM), Istanbul, Turkey

## Abstract

**Background:**

Nodal assessment in gallbladder cancer remains challenging, particularly in incidental gallbladder cancer. This understages the number of patients with node-positive disease, resulting in prognostic inaccuracy and insufficient adjuvant treatment. This study aimed to identify risk factors for positive nodes in gallbladder cancer and to compare prognostic discrimination of available nodal staging parameters.

**Methods:**

This international cohort study assessed gallbladder cancer resections undertaken between 1 January 2010 and 31 December 2020. Logistic regression was used to identify risk factors for node-positive status and develop a risk prediction score for positive nodes. Nodal staging models, including nodal site, number of positive nodes, and positive node ratio were compared for greatest prognostic discrimination in gallbladder cancer.

**Results:**

A total of 3676 patients underwent gallbladder cancer resection across 133 centres in 41 countries. Tumour (T) stage (T2, *P* = 0.012; T3, *P* = 0.002; and T4, *P* < 0.001), lymphovascular and perineural infiltration (*P* < 0.001), and tumour differentiation (*P* < 0.001) carried the greatest risk of positive nodes. These three parameters comprised the OMEGA Node Positivity Prediction Score (OMEGA-NOPPS) with C-statistics of 0.81 (95% confidence interval 0.78 to 0.84) in the training data set and 0.79 (0.73 to 0.85) in the test data set for identification of node-positive status, highlighting a ≥ 20% increased risk of positive nodes in poorly differentiated tumours with lymphovascular and perineural infiltration despite T1 disease.

**Conclusion:**

Data from this large multicentre study confirmed that the number of positive nodes is the most discriminative prognostic model for nodal staging in gallbladder cancer. OMEGA-NOPPS provides three simple parameters to stratify nodal involvement according to risk. Incidental gallbladder cancer with lymphovascular and perineural infiltration and poorly differentiated tumours, including early T stages, should be considered for further treatment.

## Teaser

Node-positive disease in gallbladder cancer is associated with poorer survival. This study showed that the number of positive nodes is the most discriminative nodal staging model for prognosis.

Furthermore, the OMEGA Node Positivity Prediction Score uses the three simple parameters of lymphovascular and perineural infiltration, tumour differentiation, and tumour stage to predict nodal involvement and identify patients who may need adjuvant treatment despite early-stage disease.

## Introduction

Gallbladder cancer (GBC) is rare but aggressive, and is the most common tumour of the biliary tract^1,2^. There have been no large prospective studies on surgical management of GBC, hence robust evidence for treatment strategies is lacking. Smaller studies lack applicable findings, and cancer registry data sets have insufficient granularity to affect practice.

One key aspect of staging GBC is identifying nodal involvement, which suggests systemic dissemination and indicates adjuvant treatment. The OMEGA (Operative Management of Gallbladder Cancer) international study^3^ confirmed nodal stage as a key prognostic marker associated with recurrence-free survival (RFS). Yet lymphadenectomy rates remain low despite international guidelines^4–10^, and patients with incidental GBC (histologically identified following cholecystectomy for suspected benign disease) often have lymphadenectomy omitted at initial surgery. The resulting absence of nodal assessment may understage the 3.4–20% of T1 tumours reported to have positive nodes^4,11–14^. Better stratification of the risk of nodal involvement could identify patients with early but aggressive tumours and a correspondingly higher risk of node-positive disease who would benefit from adjuvant treatment. Furthermore, there is ongoing debate^15–21^ regarding the optimal nodal parameters to guide prognosis, disputing the widely used 8th American Joint Committee on Cancer (AJCC) classification in favour of other parameters such as positive lymph node ratios (PLNRs).

In this publication, the large OMEGA study data set was investigated to identify the optimal prognostic nodal parameter and to develop a risk score for nodal involvement in GBC.

## Methods

### Ethical approval

Ethical approval was obtained from the Research and Development Office at Cambridge University Hospitals National Health Service Foundation Trust (the lead site) and the UK Research Ethics Committee (IRAS ID 285918). Other participating centres obtained further approvals as needed. This study was conducted and reported in compliance with the STROBE guidelines^18^ for cohort studies.

### Collaborating centres

Collaborating centres were recruited to the OMEGA study by invitation disseminated via e-mails to all members of the three international hepatopancreatobiliary (HPB) associations (the European–African HPB Association, the Americas HPB Association, and the Asia-Pacific HPB Association).

#### Inclusion and exclusion criteria

Patients were included in this study if they had undergone surgery between 1 January 2010 and 31 December 2020 for GBC that was either diagnosed before surgery or incidental (identified following cholecystectomy for benign disease). Exclusion criteria were high-grade dysplasia only, metastatic disease at surgery, or macroscopic tumour remaining at the end of resection (R2).

#### Demographic and pathological data collection

Clinical parameters, operative details, pathological findings, and follow-up and survival data were obtained from institutional databases. Co-morbidities were incorporated into the Charlson Co-morbidity Index. Incidence of GBC was determined using Globocan 2020 data, using the top quartile (>1·1 GBC cases per 100 000 population) as the threshold for “high” incidence on multivariable analyses.

#### Operative details

Extent of surgery was defined as cholecystectomy only, wedge resection (taking the liver margin at the gallbladder bed), resection of liver segments IVb and V, or major hepatectomy (right, extended left, or extended right hemihepatectomy). Data were also obtained on specific nodal groups resected, positive lymph node number (PLNN), total lymph nodes excised (TLNE), extrahepatic bile duct resection (EBDR), resection of additional organs, and surgical approach. Complications within 30 days after surgery were classified using the Clavien–Dindo scale^22^. Histology was recorded using the Eighth AJCC classification (2018) for GBC^23^, incorporating parameters such as differentiation status and lymphovascular or perineural invasion (LVPI).

#### Follow-up and survival

RFS was defined as the interval between date of surgery and date of first recurrence on imaging.

### Statistical analysis

Factors associated with TLNE were analysed using Poisson and negative binomial regression models and data expressed as risk ratios (RRs). Binary data were analysed using logistic regression, recursive partitioning classification trees, and random forest models. Survival data were analysed using the Cox model and Kaplan–Meier plots. Separate multivariable analyses excluding patients without lymphadenectomy were employed to assess the association of PLNR and AJCC nodal classification with RFS because of collinearity between these variables. Recursive partitioning classification was also used to select the most suitable cut-off values for PLNN and PLNR. The predictive ability and goodness-of-fit of the prognostic models were compared using the Akaike information criterion and C-statistic.

To develop the risk prediction model for node involvement, the data set was split into 80 and 20% training and test sets, and ten-fold cross-validation was repeated ten times on the training data set to select hyperparameters for the tree and random forest analyses. The predictive discrimination of the risk score was assessed on the test data set as a separate validation cohort. The final risk score was derived by multiplying the coefficients of the logistic regression model by 3, rounding to the nearest integer, then adding the transformed coefficient. No data imputation was used, and patients were included in this analysis only if data on all the parameters were available. The full statistical methodology is available in the *Supplementary material*.

## Results

The OMEGA study identified 4138 patients who had undergone surgery for GBC in 41 countries across 133 participating centres (*Fig. S1*). After excluding 78 patients with only high-grade dysplasia and 384 with metastatic disease/R2 resection, 3676 patients were finally included for analysis. Median overall survival and RFS times for the whole cohort were 51.2 (49.3, 52.8) and 35.2 (34.3, 36.9) months, respectively, with median follow-up of 45.3 (interquartile range 24.1–80.5) months^3^.

### TLNE

**Fig. 1:**
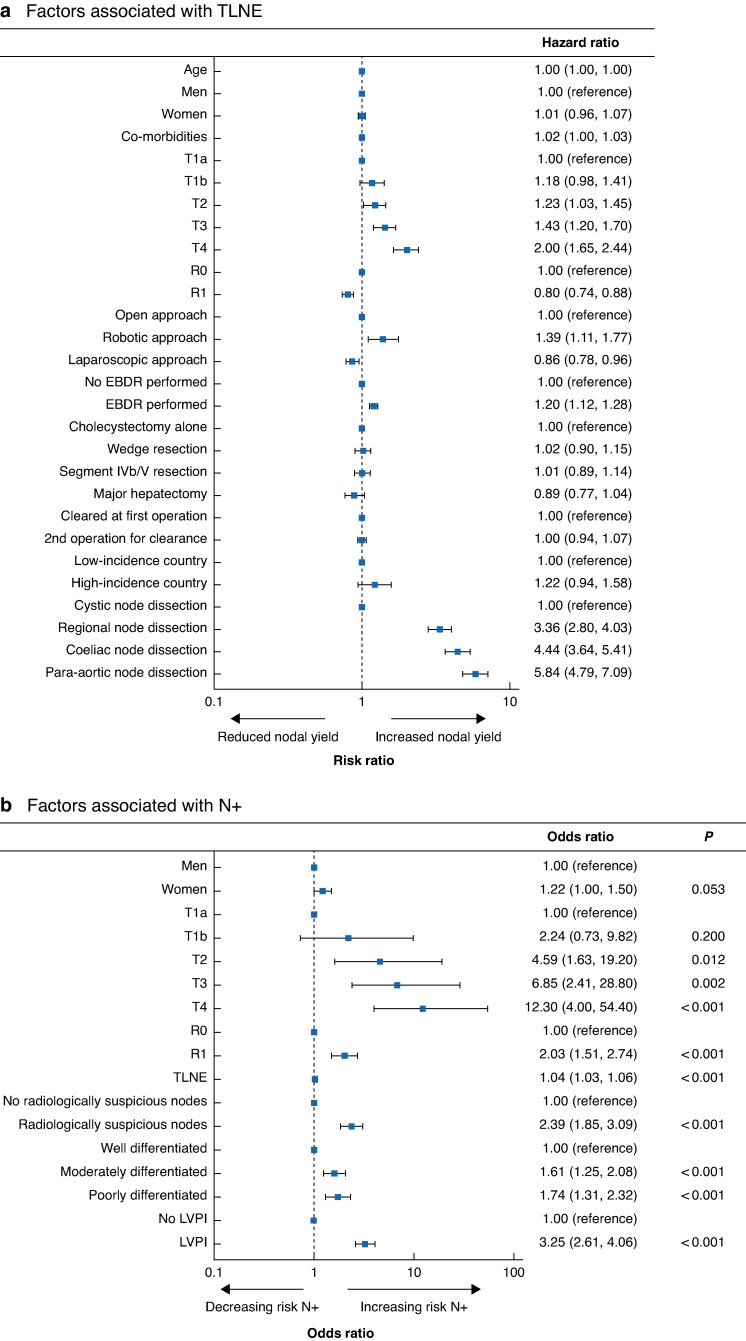
Multivariable analysis of factors associated with TLNE and of factors associated with N+ **a** Forest plot and multivariable negative binomial regression analysis of factors associated with total lymph nodes excised (TLNE), **b** Forest plot and logistic regression analysis of factors associated with positive nodes (N+). Hazard ratios and odds ratios are shown with 95% confidence intervals. EBDR, extrahepatic bile duct resection; LVPI, lymphovascular or perineural invasion.

Some 3227 patients underwent lymphadenectomy, and 1820 had ≥ 6 nodes excised. The median TLNE was 5 (interquartile range 2–9). No lymphadenectomy was undertaken in 90 (48.1%) and 75 (18.6%) patients with T1a and T1b disease, respectively. Greater TLNE was associated with T2 tumours and above compared with T1a in multivariable analysis (*Table 1* and *Fig. 1a*), and poorer TLNE with margin-positive disease (*versus* R0: RR 0.80, 95% confidence interval (c.i.) 0.74 to 0.88). Extent of liver resection showed no association with TLNE, and EBDR was associated with greater TLNE compared with no EBDR (RR 1.20, 1.12 to 1.28). A laparoscopic approach was associated with fewer TLNEs than an open approach (RR 0.86, 0.78 to 0.96), and a robotic approach was associated with more TLNEs *versus* open surgery (RR 1.39, 1.11 to 1.77). Extending nodal dissection beyond the cystic node to regional (perihilar), coeliac, and para-aortic nodes was associated with a three-, four-, and six-fold increase in TLNE, respectively, with no associated increased morbidity or mortality in a separate multivariable analysis (*Fig. S2a*). TLNE was not associated with age, sex, co-morbidities, or the need for a second operation for disease clearance (*Fig. 1a*).

**Table 1:** Demographic and clinical data according to node category

	N0	N1	N2	Nx	TLNE*
All patients (*n* = 3676)	1874 (51.0%)	1081 (29.4%)	272 (7.4%)	449 (12.2%)	5 (2–9)
**Age (years)**					
< 60 (*n* = 1044)	550 (52.7%)	316 (30.3%)	86 (8.2%)	92 (8.8%)	5 (4–6)
≥ 60 (*n* = 2632)	1324 (50.3%)	765 (29.0%)	186 (7.1%)	357 (13.6%)	6 (4–8)
**Sex**					
Male (*n* = 1252)	635 (50.7%)	361 (28.8%)	91 (7.3%)	165 (13.2%)	5 (2–9)
Female (*n* = 2424)	1239 (51.1%)	720 (29.7%)	181 (7.5%)	284 (11.7%)	5 (2–10)
**CCI score**					
< 5 (*n* = 2133)	1115 (52.2%)	646 (30.3%)	166 (7.9%)	206 (9.7%)	6 (3–10)
≥ 5 (*n* = 1321)	655 (49.6%)	376 (28.4%)	99 (7.5%)	191 (14.5%)	5 (1–9)
**Tumour differentiation**					
Well (*n* = 837)	522 (62.3%)	155 (18.5%)	27 (3.2%)	133 (15.9%)	5 (1–9)
Moderate (*n* = 1489)	731 (49.1%)	503 (33.8%)	109 (7.3%)	146 (9.8%)	5 (2–9)
Poor (*n* = 795)	305 (38.4%)	290 (36.5%)	114 (14.3%)	86 (10.8%)	6 (2–7)
**LVPI**					
Present (*n* = 1644)	591 (35.9%)	710 (43.1%)	208 (12.7%)	135 (8.2%)	6 (3–11)
Absent (*n* = 1176)	788 (67.0%)	170 (14.4%)	23 (2.0%)	195 (16.6%)	4 (1–8)
**Histology**					
T1a (*n* = 187)	92 (49.2%)	4 (2.1%)	1 (0.5%)	90 (48.1%)	1 (0–4)
T1b (*n* = 403)	294 (73.0%)	31 (7.7%)	3 (0.7%)	75 (18.6%)	4 (1–8)
T2 (*n* = 1700)	991 (58.3%)	461 (27.1%)	68 (4.0%)	180 (10.6%)	5 (2–9)
T3 (*n* = 1186)	453 (38.2%)	487 (41.1%)	151 (12.7%)	95 (8.0%)	6 (3–10)
T4 (*n* = 200)	44 (22.0%)	98 (49.0%)	49 (24.5%)	9 (4.5%)	9 (5–15)
R0 (*n* = 3187)	1748 (54.8%)	869 (27.3%)	213 (6.7%)	357 (11.2%)	5 (2–9)
R1 (*n* = 472)	120 (25.4%)	210 (44.5%)	58 (12.3%)	84 (17.8%)	4 (1–8)
**Surgical access**					
Open (*n* = 3162)	1625 (51.4%)	996 (31.5%)	261 (8.3%)	280 (8.9%)	6 (2–10)
Laparoscopic (*n* = 465)	218 (46.9%)	74 (15.9%)	9 (1.9%)	164 (35.2%)	1 (0–6)
Robotic (*n* = 36)	24 (66.7%)	9 (25.0%)	1 (2.8%)	2 (3.6%)	6 (3–11)
**EBDR**					
Yes (*n* = 976)	380 (38.9%)	412 (42.2%)	141 (14.4%)	43 (4.4%)	7 (3–12)
No (*n* = 2700)	1494 (55.3%)	669 (24.8%)	131 (4.9%)	406 (15.0%)	4 (1–8)
**Extent of resection**					
Cholecystectomy alone (*n* = 557)	197 (35.4%)	77 (13.8%)	10 (1.8%)	273 (49.0%)	0 (0–2)
Wedge resection (*n* = 1407)	817 (58.1%)	426 (30.3%)	79 (5.6%)	85 (6.0%)	6 (3–10)
Segment IVb–V resection (*n* = 1397)	755 (54.0%)	436 (31.2%)	131 (9.4%)	75 (5.4%)	6 (3–9)
Major hepatectomy (*n* = 315)	105 (33.3%)	142 (45.1%)	52 (16.5%)	16 (5.1%)	7 (3–13)
**Extent of nodal dissection**					
Cystic (*n* = 142)	100 (70.4%)	23 (16.2%)	0 (0%)	19 (13.4%)	1 (1–6)
Regional (*n* = 2332)	1286 (55.1%)	779 (33.4%)	184 (7.9%)	33 (3.6%)	6 (3–9)
Coeliac (*n* = 267)	140 (52.4%)	92 (34.4%)	33 (12.3%)	2 (0.7%)	7 (4–13)
Para-aortic (*n* = 417)	236 (56.6%)	126 (30.2%)	51 (12.2%)	4 (1.0%)	11 (7–17)
**Surgical stages**					
Single stage (*n* = 2139)	882 (41.2%)	692 (32.3%)	196 (9.2%)	369 (17.3%)	5 (1–9)
Two stage (*n* = 1537)	992 (64.5%)	389 (25.3%)	76 (4.9%)	80 (5.2%)	5 (3–9)
**Incidence of GBC**					
High (*n* = 1209)	638 (52.8%)	371 (30.7%)	96 (7.9%)	104 (8.6%)	8 (4–12)
Low (*n* = 2467)	1236 (50.1%)	710 (28.8%)	176 (7.1%)	345 (14.0%)	4 (1–8)

*Values are median (interquartile range). TLNE, total lymph nodes excised; CCI, Charlson Co-morbidity Index; LVPI, lymphovascular or perineural invasion; EBDR, extrahepatic bile duct resection.

### Tumour (T) stage

Positive lymph nodes (N+ disease) were identified in 1353 patients across the entire cohort (*Table 1*). Some 1081 patients had N1 disease (≤ 3 positive nodes), and 272 had N2 disease (> 3 positive nodes). N+ disease was predominantly associated with higher T stages in multivariable analysis, but was also present in 5 patients (2.6%) with T1a and 34 (8.4%) with T1b tumours, *Table 1* and *Fig. 1b*). Poorly differentiated tumours (odds ratio (OR) 1.74, 95% c.i. 1.31 to 2.32; *P* < 0.001) and LVPI (OR 3.25, 2.61 to 4.06; *P* < 0.001) were also associated with N+ disease. Suspected nodal involvement on computed tomography (CT), magnetic resonance imaging, or positron emission tomography–CT before surgery was also significantly associated with N+ status (OR 2.39, 1.85 to 3.09; *P* < 0.001). TLNE had a small but significant association with N+ disease (OR 1.04, 1.03 to 1.06; *P* < 0.001). Non-regional node dissection was not associated with N+ disease (*Fig. S2b*).

### Prognostic association with RFS

**Fig. 2:**
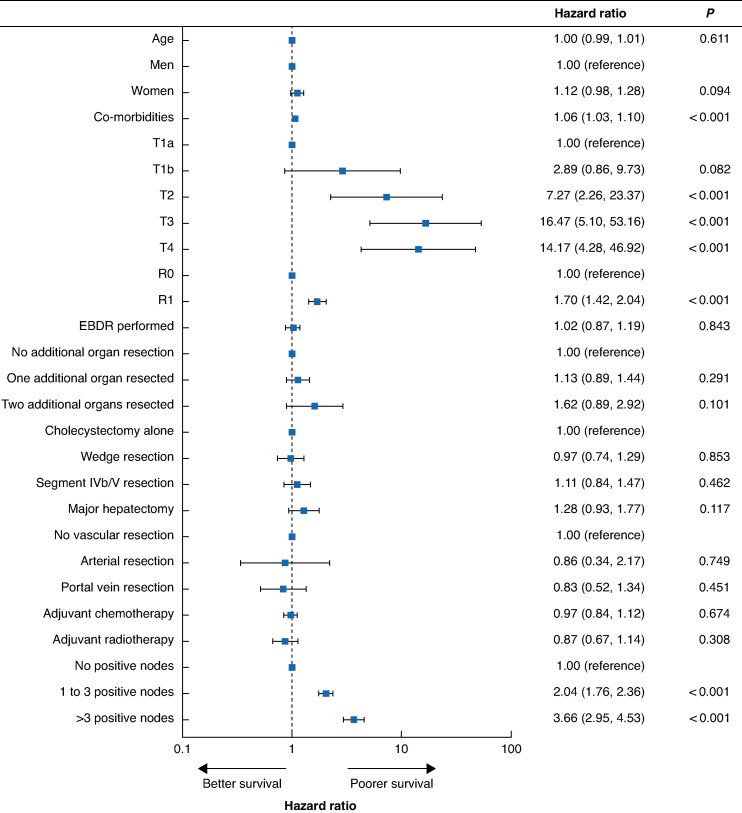
Multivariable analysis of factors associated with recurrence-free survival Forest plot and multivariable Cox proportional hazard regression analysis of factors associated with recurrence-free survival. Hazard ratios are shown with 95% confidence intervals. EBDR, extrahepatic bile duct resection.

Higher T stages, margin-positive disease, and N+ status had the greatest association with RFS in multivariable analysis (*Fig. 2*). Non-regional node dissection and TLNE had no significant effect on RFS (*Fig. S3a*,*b*). Survival recursive partitioning tree models identified the PLNN of three nodes as the most discriminatory cut-off for RFS, and was associated with worse RFS in multivariable analysis (*P* < 0.001) (*Fig. 2* and *Fig. S4a*) (*Table 2*). N+ status in any nodal group was associated with worse RFS compared with N0 disease, particularly in para-aortic nodes (*P* < 0.001) (*Fig. S5a*).

**Table 2:** Discriminative ability of prognostic models for node categorization

Prognostic model	AIC	C-index*	Model threshold	Hazard ratio†‡	*P*
PLNR continuous	14720.27	0.7581(0.0069)	–	3.00 (2.44, 3.69)	< 0.001
PLNR categorical (> 0.1)	14692.51	0.7615(0.0068)	PLNR ≤ 0.1	1.00 (reference)	
			PLNR > 0.1	2.22 (1.93, 2.55)	< 0.001
AJCC 7th edition	14694.96	0.7603(0.0069)	N0	1.00 (reference)	
			N1	2.19 (1.92, 2.52)	< 0.001
			N2	2.64 (1.90, 3.67)	< 0.001
AJCC 8th edition	14660.80	0.7633(0.0068)	N0	1.00 (reference)	
			N1	2.04 (1.76, 2.36)	< 0.001
			N2	3.66 (2.95, 4.53)	< 0.001

Values in parentheses are *standard errors and †95% confidence intervals. ‡Imported from individual multivariable analyses using each of the prognostic models, as in *Figs S2* and *S3*. AIC, Akaike information criterion; PLNR, positive lymph node ratio; AJCC, American Joint Committee on Cancer.

Using the same recursive partitioning methodology as above, a threshold of > 0.1 was identified as the best discriminator of RFS for PLNR, and was associated with worse RFS in multivariable analysis (*P* < 0.001) (*Figs S4c* and *S5c*). PLNR was associated with a three-fold hazard to RFS when assessed as a continuous variable (*P* < 0.001) (*Fig. S5d*).

### Scoring system for N+ status in incidental GBC

The three parameters chosen for the OMEGA Node Positivity Prediction Score (OMEGA-NOPPS), namely T category, tumour differentiation, and LVPI, had all exhibited significant association with N+ status and are available routinely for patients with incidental GBC (*Table 3*) (*Fig. S6*). This analysis utilized 1141 individuals from the main cohort for whom the relevant data were available, split into 80 : 20 training : test data sets. The train data set (912 patients) exhibited a C-statistic of 0.81 (95% c.i. 0.78 to 0.84), sensitivity of 70.6%, and specificity of 76.9%. The separate test or validation cohort (229 patients) showed good generalizability of the model, with a C-statistic of 0.79 (0.73 to 0.85), sensitivity of 72.3%, and specificity of 74.8%. Calibration curves showed agreement between predicted and observed outcomes, without signs of overfitting or underfitting (*Fig. S7*). Notably, OMEGA-NOPPS quantifies the additional risk conferred by tumour differentiation status and LVPI on top of T stage.

**Table 3:** OMEGA Node Positivity Prediction Score stratification for nodal involvement in gallbladder cancer

	Score
**Tumour (T) stage**	
T1a	0
T1b	3
T2	5
T3	7
T4	10
**Tumour differentiation**	
Well	0
Moderate	1
Poor	2
**LVPI**	
No	0
Yes	4
**Final risk score**	**Probability of positive nodes (%)**
0–2	≤ 6.5
2–4	6.5–11.9
4–6	11.9–20.9
6–8	20.9–33.9
8–10	33.9–50.0
10–12	50.0–66.1
12–14	66.1–79.1
14–16	79.1–88.1

As an example, a patient with a T1b tumour would be expected to have an 11.9% risk of postive nodes if they had a well differentiated tumour without lymphovascular or perineural invasion (LVPI), but a 50% risk with a poorly differentiated tumour and LVPI.

## Discussion

In this multicentre study, T stage, differentiation, and LVPI were identified as key risk factors for node-positive disease, and number of positive nodes was confirmed as the most important prognostic nodal factor for RFS.

This study showed that coeliac and para-aortic nodal dissection could be undertaken safely, although good nodal yields can be obtained with regional lymphadenectomy alone. EBDR was associated with more lymph nodes retrieved, in keeping with some studies; however, increasing data^7,24–27^ indicate that good TLNEs can still be obtained without EBDR, which is no longer recommended routinely in GBC resection because of increased morbidity.

The eighth AJCC classification was introduced in 2018, stratifying GBC nodal stage by PLNN rather than nodal site, but subsequent studies^16,18,23,28–32^ have queried the value of the eighth AJCC classification compared with other staging parameters such as PLNR. The eighth AJCC classification had a small but clear advantage over the other parameters in this study, which, unlike previous studies^17,18,28,31,32^, combines a large cohort with the necessary granularity and a high lymphadenectomy rate. In contrast to other nodal staging prognostic models, the eighth AJCC classification requires no complex calculations and is widely used in histopathological reporting internationally. This makes it feasible, practical, and easily reproducible for prognostication of node-positive disease and decisions regarding adjuvant treatment.

This study showed that poorly differentiated tumours with LVPI would carry a ≥ 20% risk of node-positive disease. Cholecystectomy alone is increasingly considered sufficient treatment for T1 tumours; however, as many as 10% of such patients could harbour positive nodes, overlooked without a lymphadenectomy, and thus not receive adjuvant treatment^6–10,12,14,33^. Therefore, OMEGA-NOPPS informs consideration of further surgery for early-stage tumours, and, for higher scores, may allow consideration of adjuvant treatment without the cost and morbidity of confirmatory lymphadenectomy. Given the promise offered by ongoing adjuvant and neoadjuvant chemotherapy and immunotherapy trials in GBC^34–37^, this is a key opportunity to maximize benefit in a group of patients for whom long-term survival may be possible.

The three parameters in OMEGA-NOPPS are routine histopathological parameters mandated by international histopathological reporting guidelines^38,39^, readily available in incidental GBC, and easily incorporated into tumour board discussions. Additionally, the use of parameters beyond T stage may counterbalance any inadvertent understaging of tumour extent in cholecystectomy specimens. OMEGA-NOPPS complements existing scores for GBC recurrence, survival, and adjuvant treatment, facilitating more personalized prognostication and management^40–42^. Importantly, the wide global population within the present study should render this score internationally applicable in both high- and low-incidence areas, and allow further validation in a prospective multicentre setting.

The main strengths of this study are the large numbers of patients and centres recruited, which allows a truly global picture of the practice of GBC surgery, with a high lymphadenectomy rate and incorporating a level of detail not available in single-centre cancer registries. Nevertheless, this study has a number of limitations. Given the retrospective nature of the study, specific data on the number of nodes retrieved and exact nodal sites were not always available, and the existence of anatomically continuous nodal chains rendered further detailed breakdown of adjacent nodal subgroups difficult. Lymphadenectomy rates for T1a and T1b disease in this study were much higher than in other studies^10,43^, particularly compared with registry-based data where nodal staging was absent in almost half of patients with T1b disease. This may be because of increased lymphadenectomy rates in recent years, but may also represent differences in treatment strategies as well as overall survival and RFS rates between tertiary HPB centres in this study and the more heterogeneous data pool in national registries^44^. The exact reason for initial cholecystectomy was not always known, so it was not possible to analyse patients with incidental early-stage GBC separately. OMEGA-NOPPS relies on pathological variables only available after surgery in patients with incidental GBC. Improved imaging techniques and novel molecular biomarkers are necessary for better prediction of nodal metastases for GBC detected before surgery, and are thus a key area for future research.

The data from this study showed that identification of four or more positive nodes has better prognostic discrimination than nodal ratio or more distant positive nodes, highlighting the importance of a thorough regional lymphadenectomy to accurately stage GBC. The new OMEGA-NOPPS model provides a simple three-parameter score to risk-stratify nodal involvement and highlight patients with LVPI and poorly differentiated incidental GBC who may otherwise be overlooked for further treatment, ensuring a more nuanced discussion and personalized treatment of this rare and aggressive tumour.

## Collaborators

Tomoyuki Abe (Onomichi General Hospital, Hiroshima, Japan), Moh'd Abu Hilal (Poliambulanza Foundation Hospital, Brescia, Italy), Maria del Mar Achalandabaso Boira (Hospital Universitario Vall d´Hebron, Barcelona, Spain), Mustapha Adham (Edouard Herriot Hospital, Lyon, France), Mohamed Adam (University of California San Francisco, California, USA), Maryam Ahmad (Emory University, Atlanta, Georgia, USA), Bilal Al-Sarireh (Morriston Hospital, Swansea, United Kingdom), Maite Albiol (Hospital Universitari Girona Dr Josep Trueta, Girona, Spain), Nassir Alhaboob (Soba University Hospital, Khartoum, Sudan), Adnan Alseidi (University of California San Francisco, California, USA), Houssem Ammar (Sahloul Hospital, Tunisia), Akshay Anand (King George's Medical University, Lucknow, India), Bodil Andersson (Skane University Hospital, Lund, Sweden), Pantelis Antonakis (Aretaieion University Hospital, Athens, Greece), Veronica Araya (Hospital Sotero del Rio and Pontificia Catolica Universidad de Chile, Santiago, Chile), Stanley W Ashley (Brigham and Women's Hospital, Boston, USA), Georgi Atanasov (Royal Adelaide Hospital, Adelaide, Australia), Fabio Ausania (Hospital Clinic Barcelona, Barcelona, Spain), Ricardo Balestri (Azienda Ospedaliero Universitaria Pisana, Pisa, Italy), Abhirup Banerjee (Royal Free Hospital, London, United Kingdom), Sudeep Banerjee (Tata Medical Centre, Kolkata, India), Simon Banting (Melbourne Surgical Group, Melbourne, Australia), Giedrius Barauskas (Hospital of Lithuanian University of Health Sciences Kauno Klinikos, Kaunas, Lithuania), Fabian Bartsch (Universitätsmedizin Mainz, Mainz, Germany), Andrea Belli (National Cancer Instititute G Pascale, Napoli, Italy), Simona Beretta (ASST Grande Ospedale Metropolitano Niguarda, Italy), Frederik Berrevoet (University Hospital Ghent, Ghent, Belgium), Ramesh Singh Bhandari (Tribhuvan University Teaching Hospital, Kathmandu, Nepal), Gerardo Blanco Fernandez (University of Extremadura, Badajoz, Spain), Louisa Bolm (University Medical Center, Lübeck, Germany), Mathieu Bonal (Croix Rousse University Hospital , Lyon, France), Emre Bozkurt (Koc University Hospital, Istanbul, Turkey), Andries E Braat (Leiden University Medical Center, Leiden, Netherlands), Luke Bradshaw (St Vincent's Hospital, Melbourne, Australia), Konstantinos Bramis (Aretaieion University Hospital, Athens, Greece), Alejandro Branes (Hospital Sotero del Rio and Pontificia Catolica Universidad de Chile, Santiago, Chile), Lyle Burdine (University of Arkansas for Medical Sciences, Arkansas, USA), Matthew Byrne (University of Rochester Medical College, New York, USA), Maria Caceres (Dos de Mayo Hospital, Lima, Peru), Maria Jesus Castro Santiago (Hospital Universitario Puerta del Mar, Cadiz, Spain), Benjamin Chan (Royal Liverpool University Hospital, Liverpool, United Kingdom), Lynn Chong (St Vincent's Hospital, Melbourne, Australia), Ahmet Çoker (Izmir HPB Clinic, Izmir, Turkey), Maria Conde Rodriguez (Hospital Universitario Lucus Augusti, Lugo, Spain), Daniel Croagh (Monash Hospital, Melbourne, Australia), Alyn Crutchley (Leeds Teaching Hospitals NHS Trust, Leeds, United Kingdom), Carmen Cutolo (National Cancer Instititute G Pascale, Napoli, Italy), Mathieu D'Hondt (AZ Groeninge, Kortrijk, Belgium), Daniel D'Souza (McMaster University, Hamilton, Ontario, Canada), Freek Daams (Amsterdam UMC, Netherlands), Raffaele Dalla Valle (Parma University Hospital, Parma, Italy), José Davide (Centro Hospitalar Universitário do Porto, Porto, Portugal), Mario de Bellis (University of Verona Medical School, Verona, Italy), Marieke de Boer (University Medical Center Groningen, Netherlands), Celine de Meyere (AZ Groeninge, Kortrijk, Belgium), Philip de Reuver (Radboud UMC, Nijmegen, Netherlands), Matthew Dixon (Penn State College of Medicine, Pennsylvania, USA), Panagiotis Dorovinis (Laiko General Hospital and Kapodistrian University of Athens, Greece), Gabriela Echeverría Bauer (Clinical Alemana, Santiago, Chile), Maria Eduarda (Federal University of Rio de Janeiro, Rio de Janeiro, Brazil), Hasan Eker (University Hospital Ghent, Ghent, Belgium), Joris Erdmann (Amsterdam UMC, Netherlands), Mert Erkan (Koc University Hospital, Istanbul, Turkey), Evangelos Felekouras (Laiko General Hospital and Kapodistrian University of Athens, Greece), Emanuele Felli (Nouvel Hospital Civil, Strasbourg, France), Eduardo Fernandes (Federal University of Rio de Janeiro, Rio de Janeiro, Brazil), Eduardo Figueroa Rivera (Hospital Clínico Regional Dr Guillermo Grant Benavente, Concepcion, Chile), Andras Fulop (Semmelweis University, Budapest, Hungary), Daniel Galun (University Clinical Center of Serbia, Belgrade, Serbia), Michael Gerhards (OLVG, Amsterdam, Netherlands), Poya Ghorbani (Karolinska University Hospital, Stockholm, Sweden), Fabio Giannone (Nouvel Hospital Civil, Strasbourg, France), Luis Gil (University of Buenos Aires, Beunos Aires, Argentina), Emmanouil Giorgakis (University of Arkansas for Medical Sciences, Arkansas, USA), Mario Giuffrida (Parma University Hospital, Parma, Italy), Felice Giuliante (Fondazione Policlinico Universitario A. Gemelli, Rome, Italy), Ioannis Gkekas (Kirurgcentrum, Umea University, Umea, Sweden), Miguel Gomez Bravo (Hospital Virgen Del Rocio, Spain), Bas Groot Koerkamp (Erasmus MC, Rotterdam, Netherlands), Oscar Guevara (Colombian National University, Bogota, Colombia), Alfredo Guglielmi (University of Verona Medical School, Verona, Italy), Aiste Gulla (Vilnius University Hospital, Vilnius, Lithuania), Rahul Gupta (Synergy Institute of Medical Sciences, Dehradun, India), Amit Gupta (All India Institute of Medical Sciences, Rishikesh, India), Marta Gutiérrez (Hospital Miguel Servet, Zaragoza, Spain), Abu Bakar Hafeez Bhatti (Shifa International Hospital, Islamabad, Pakistan), Jeroen Hagendoorn (UMC Utrecht Cancer Center, Utrecht, Netherlands), Zain Hajee (Manchester Foundation NHS Trust, Manchester, United Kingdom), Abdul Rahman Hakeem (Leeds Teaching Hospitals NHS Trust, Leeds, United Kingdom), Hytham Hamid (Soba University Hospital, Khartoum, Sudan), Sayed Hassen (Eastern Health Box Hill, Australia), Stefan Heinrich (Universitätsmedizin Mainz, Mainz, Germany), Roberto Hernandez-Alejandro (University of Rochester Medical College, New York, USA), Ryota Higuchi (Tokyo Women's Medical Hospital, Tokyo, Japan), Daniel Hoffman (University of California San Francisco, California, USA), David Holroyd (Glasgow Royal Infirmary, Glasgow, United Kingdom), Daniel Hughes (Oxford University Hospitals NHS Foundation Trust, Oxford, United Kingdom), Arpad Ivanecz (University Medical Center Maribor, Maribor, Slovenia), Satheesh Iype (Royal Free Hospital, London, United Kingdom), Isabel Jaen Torrejimeno (University of Extremadura, Badajoz, Spain), Shantanu Joglekar (Eastern Health Box Hill, Australia), Robert Jones (Royal Liverpool University Hospital, Liverpool, United Kingdom), Klaus Kaczirek (University of Medicine Vienna, Austria), Harsh Kanhere (Royal Adelaide Hospital, Adelaide, Australia), Ambareen Kausar (Royal Blackburn Hospital, Blackburn, United Kingdom), Zhanyi Kee (Hospital Sultanah Bahiyah, Alor Setar, Malaysia), Jessica Keilson (Emory University, Atlanta, Georgia, USA), Jorg Kleef (University Medicine Halle, Halle, Germany), Johannes Klose (University Medicine Halle, Halle, Germany), Brett Knowles (Royal Melbourne Hospital, Melbourne, Australia), Jun Kit Koong (University Malaya Medical Centre, Kuala Lumpur, Malaysia), Nagappan Kumar (University Hospital of Wales, Cardiff, United Kingdom), Supreeth Kunnuru (Royal Free Hospital, London, United Kingdom), Paleswan Joshi Lakhey (Tribhuvan University Teaching Hospital, Kathmandu, Nepal), Andrea Laurenzi (University of Bologna, Bologna, Italy), Yeong Sing Lee (University Malaya Medical Centre, Kuala Lumpur, Malaysia), Felipe Leon (Hospital Clinic Barcelona, Barcelona, Spain), Voon Meng Leow (Hospital Sultanah Bahiyah, Alor Setar, Malaysia), Jean-Baptiste Lequeu (Dijon University Hospital, Dijon, France), Mickael Lesurtel (Croix Rousse University Hospital, Lyon, France), Elisabeth Lo (McMaster University, Hamilton, Ontario, Canada), Stefan Löb (University Hospital Würzburg, Würzburg, Germany), Elizabeth Lockie (Royal Melbourne Hospital, Melbourne, Australia), Peter Lodge (Leeds Teaching Hospitals NHS Trust, Leeds, United Kingdom), Dolores López Garnica (Hospital Universitario Vall d´Hebron, Barcelona, Spain), Victor Lopez Lopez (Clinic and University Virgen de la Arrixaca Hospital, Murcia, Spain), Linda Lundgren (University Hospital Linkoping, Sweden), Nikolaos Machairas (Laiko General Hospital and Kapodistrian University of Athens, Greece), Dhiresh Maharjan (Kathmandu Medical College, Nepal), Deep Malde (Leicester Hospitals, Leicester, United Kingdom), Marc Mankarious (Penn State College of Medicine, Pennsylvania, USA), Guillaume Martel (University of Ottawa, Ottawa, Canada), Julie Martin (University of South Carolina, South Carolina, USA), Michele Mazzola (ASST Grande Ospedale Metropolitano Niguarda, Italy), Arianeb Mehrabi (Ruprecht-Karls University, Heidelberg, Germany), Ricardo Memeo (Regional General Hospital F. Miulli, Bari, Italy), Flavio Milana (Humanitas Hospital, Milan, Italy), George Molina (Brigham and Women's Hospital, Boston, USA), Leah Monette (University of Ottawa, Ottawa, Canada), Haluk Morgul (University of Münster, Münster, Germany), Dimitrios Moris (Laiko General Hospital and Kapodistrian University of Athens, Greece), Antonios Morsi-Yeroyannis (Ippokratio General Hospital and University Clinic Thessaloiniki, Thessaloniki, Greece), Nicholas Mowbray (University Hospital of Wales, Cardiff, United Kingdom), Francesk Mulita (University of Patras, Rion, Greece), Edoardo Maria Muttillo ( Sapienza University of Rome, Italy), Malith Nandasena (Colombo South Teaching Hospital, Colombo, Sri Lanka), Pueya Rashid Nashidengo (Windhoek Central Hospital, Windhoek, Namibia), Arash Nickkholgh (Ruprecht-Karls University, Heidelberg, Germany), Colin Byron Noel (Universitas Academic Hospital, Bloemfontein, South Africa), Masayuki Ohtsuka (Chiba University, Chiba, Japan), Arturs Ozolins (Pauls Stradina Clinical University Hospital, Riga, Latvia), Sanjay Pandanaboyana (Newcastle University Hospitals, Newcastle, United Kingdom), Nikolaos Pararas (Dr Sulaiman Al Habib Hospital, Riyadh, Saudi Arabia), Alessandro Parente (University Hospitals Birmingham, Birmingham, United Kingdom), June Peng (Penn State College of Medicine, Pennsylvania, USA), Arkaitz Perfecto Valero (Cruces University Hospital, Barakaldo, Spain), Julie Perinel (Edouard Herriot Hospital, Lyon, France), Konstatinos Perivoliotis (University Hospital of Larissa, Larissa, Greece), Teresa Perra (AOU Sassari, Sardinia, Italy), Patrick Pessaux (Nouvel Hospital Civil, Strasbourg, France), Natalie Petruch (University Medical Center, Lübeck, Germany), Gaetano Piccolo ( San Paolo Hospital, University of Milan, Milan, Italy), Laszlo Piros (Semmelweis University, Budapest, Hungary), Alberto Porcu (AOU Sassari, Sardinia, Italy), Viswakumar Prabakaran (Newcastle University Hospitals, Newcastle, United Kingdom), Raj Prasad (Leeds Teaching Hospitals NHS Trust, Leeds, United Kingdom), Mikel Prieto Calvo (Cruces University Hospital, Barakaldo, Spain), Florian Primavesi (Salzkammergut Klinikum, Vöcklabruck, Austria), Eva Maria Pueyo Periz (Hospital Virgen Del Rocio, Spain), Alberto Quaglia (Royal Free Hospital, London, United Kingdom), Jose M Ramia Angel (General Universitario de Alicante, Alicante, Spain), Ashwin Rammohan (Rela Hospital, Chennai, India), Francesco Razionale (Fondazione Policlinico Universitario A. Gemelli, Rome, Italy), Ricardo Robles Campos (Clinic and University Virgen de la Arrixaca Hospital, Murcia, Spain), Manas Roy (Tata Medical Centre, Kolkata, India), Sophie Rozwadowski (University Hospital Bristol, Bristol, United Kingdom), Luis Ruffolo (University of Rochester Medical College, New York, USA), Natalia Ruiz (Hospital Clínico Regional Dr Guillermo Grant Benavente, Concepcion, Chile), Andrea Ruzzenante (University of Verona Medical School, Verona, Italy), Lily Saadat (Brigham and Women's Hospital, Boston, USA), Mohamed Amine Said (Sahloul Hospital, Tunisia), Edoardo Saladino (AO Papardo, Messina, Italy), Gabriel Saliba (Karolinska University Hospital, Stockholm, Sweden), Per Sandstrom (University Hospital Linkoping, Sweden), Carlo Alberto Schena (Regional General Hospital F. Miulli, Bari, Italy), Anthony Scholer (University of South Carolina, South Carolina, USA), Christoph Schwarz (University of Medicine Vienna, Austria), Lorenzo Serafini (University of Bologna, Bologna, Italy), Leyre Serrablo (Hospital Miguel Servet, Zaragoza, Spain), Pablo E Serrano (McMaster University, Hamilton, Ontario, Canada), Deepak Sharma (Tribhuvan University Teaching Hospital, Kathmandu, Nepal), Aali Sheen (Manchester Foundation NHS Trust, Manchester, United Kingdom), Vishwanath Siddagangaiah (Royal Blackburn Hospital, Blackburn, United Kingdom), Michael Silva (Oxford University Hospitals NHS Foundation Trust, Oxford, United Kingdom), Saurabh Singh (King George's Medical University, Lucknow, India), Ajith Siriwardena (Manchester Foundation NHS Trust, Manchester, United Kingdom), Michal Skalski (Medical University of Warsaw, Warsaw, Poland), Mante Smig (Vilnius University Hospital, Vilnius, Lithuania), Faris Soliman (Morriston Hospital, Swansea, United Kingdom), Abhinav Arun Sonkar (King George's Medical University, Lucknow, India), Donzília Sousa Silva (Centro Hospitalar Universitário do Porto, Porto, Portugal), Ernesto Sparrelid (Karolinska University Hospital, Stockholm, Sweden), Harry V M Spiers (Cambridge University Hospitals, Cambridge, United Kingdom), Parthi Srinivasan (Kings College London, London, United Kingdom), Malin Sternby Eilard (Sahlgrenska University Hospital, Gothenburg, Sweden), Oliver Strobel (University of Medicine Vienna, Austria), Urban Stupan (University Medical Centre Ljubljana, Ljubljana, Slovenia), Miguel Angel Suarez-Munoz (University Hospital Virgen de la Victoria, Malaga, Spain), Manisekar Subramaniam (Hospital Sultanah Bahiyah, Alor Setar, Malaysia), Teiichi Sugiura (Shizuoka Cancer Centre, Shizuoka, Japan), Robert Sutcliffe (University Hospitals Birmingham, Birmingham, United Kingdom), Hilko Swank (OLVG, Amsterdam, Netherlands), Shibojit Talukder (Cambridge University Hospitals, Cambridge, United Kingdom), Lillian Taylor (Melbourne Surgical Group, Melbourne, Australia), Prabin Bikram Thapa (Kathmandu Medical College, Nepal), Catherine Teh (National Kidney and Transplant Institute, Manila, Philippines), Asara Thepbunchonchai (Rajavithi Hospital, Bangkok, Thailand), Caman Thieu (McMaster University, Hamilton, Ontario, Canada), Navneet Tiwari (Newcastle University Hospitals, Newcastle, United Kingdom), Guido Torzilli (Humanitas Hospital, Milan, Italy), Chutwichai Tovikkai (Mahidol University, Bangkok, Thailand), Blaz Trotovsek (University Medical Centre Ljubljana, Ljubljana, Slovenia), Savvas Tsaramanidis (Ippokratio General Hospital and University Clinic Thessaloiniki, Thessaloniki, Greece), Georgios Tsoulfas (Ippokratio General Hospital and University Clinic Thessaloiniki, Thessaloniki, Greece), Katsuhiko Uesaka (Shizuoka Cancer Centre, Shizuoka, Japan), Garzali Umar (Aminu Kano Teaching Hospital, Kano, Nigeria), Lucio Urbani (Azienda Ospedaliero Universitaria Pisana, Pisa, Italy), Michail Vailas (University of Patras, Rion, Greece), Ronald van Dam (MUMC Maastricht, Maastricht, Netherlands), Peter van de Boezem (Radboud UMC, Nijmegen, Netherlands), Stijn van Laarhoven (University Hospital Bristol, Bristol, United Kingdom), Tomas Vanagas (Hospital of Lithuanian University of Health Sciences Kauno Klinikos, Kaunas, Lithuania), Mike Van Dooren (Radboud UMC, Nijmegen, Netherlands), Manon Viennet (Dijon University Hospital, Dijon, France), Luca Vigano (Humanitas Hospital, Milan, Italy), Aarathi Vijayashanker (Kings College London, London, United Kingdom), Celia Villodre (General Universitario de Alicante, Alicante, Spain), Toshifumi Wakai (Niigata University, Niigata, Japan), Aklile Workneh (University of Ottawa, Ottawa, Canada), Li Xu (China Japan Friendship Hospital, Beijing, China), Masakazu Yamamoto (Tokyo Women's Medical Hospital, Tokyo, Japan), Zhiying Yang (China Japan Friendship Hospital, Beijing, China), Robert Young (Royal Liverpool University Hospital, Liverpool, United Kingdom), Marko Zivanovic (University Clinical Center of Serbia, Belgrade, Serbia).

## Supplementary Material

zraf056_Supplementary_Data

## Data Availability

The data underlying this article may be shared on reasonable request to the corresponding author.
