# Supplementary material for: Assessment of nodal staging and risk factors for nodal involvement in gallbladder cancer
Source: BJS Open. 2025 May 23;9(3):zraf056. doi: 10.1093/bjsopen/zraf056 (PMC12100740; doi:10.1093/bjsopen/zraf056)
Supplement: zraf056_Supplementary_Data [file zraf056_supplementary_data.docx]

**Assessment of nodal staging and risk factors for nodal involvement in gallbladder cancer: retrospective study**

Anita Balakrishnan PhD FRCS^a^, Petros Barmpounakis PhD^b^, Nikolaos Demiris PhD^b^, Bodil Andersson MD PhD^c^, Alejandro Brañes MD^d^, Xavier de Aretxabala MD^e^, Malin Sternby Eilard MD PhD^f^, Paul Gibbs MD FRCS^a^, Simon J F Harper MD FRCS^a^, Emmanuel L Huguet PhD FRCS^a^, Asif Jah FRCS^a^, Vasilis Kosmoliaptsis PhD FRCS^a^, Javier Lendoire MD PhD^g^, Siong S Liau MD FRCS^a^, Shishir Maithel MD^h^, Jack L Martin PhD FRCS^a^, Colin Noel MD, MMed^i^, Raaj K Praseedom FRCS^a^, Alejandro Serrablo MD PhD^j^, Volkan Adsay MD^k^ for the OMEGA study investigators

^a^Department of HPB Surgery, Cambridge University Hospitals NHS Foundation Trust, and Department of Surgery, University of Cambridge, Hills Road, Cambridge, CB2 0QQ, United Kingdom

^b^Cambridge Clinical Trials Unit – Cancer Theme, Cambridge University Hospitals NHS Foundation Trust, Hills Road, Cambridge, CB2 0QQ, United Kingdom, and Department of Statistics, Athens University of Economics and Business, Athens, Greece

^c^Department of Surgery, Lund University, Skane University Hospital, Lund, Sweden

^d^Department of HPB Surgery, Hospital Sotero del Rio, Av. Concha y Toro 3459, Puente Alto, Región Metropolitana, Chile

^e^Department of Digestive Surgery, Hepato-Pancreato-Biliary Surgery Unit, Surgery Service, Gallbladder Consortium Chile, Sotero del Rio Hospital and Clinica Alemana, Santiago, Chile

^f^ Transplantation Center, Sahlgrenska University Hospital and Department of Surgery, Institute of Clinical Sciences, Sahlgrenska Academy, University of Gothenburg, Gothenburg, Sweden

^g^Department of Surgery, University of Buenos Aires, Hospital Dr Cosme Argerich, Buenos Aires, Argentina

^h^Department of Surgery, Emory University School of Medicine, Atlanta, Georgia, 30322 United States of America

^i^Gastrointestinal Surgery and HPB Surgery, Department of Surgery, University of the Free State, Bloemfontein, South Africa

^j^Department of HPB Surgery, Miguel Servet University Hospital, Zaragoza, Spain

^k^Department of Pathology, Koç University Hospital, Istanbul 34010, Turkey; Koç University Research Center for Translational Medicine (KUTTAM), Istanbul 34010, Turkey

Corresponding author:

Anita Balakrishnan, PhD FRCS

Consultant Hepatopancreatobiliary Surgeon, Department of HPB Surgery, Cambridge University Hospitals NHS Foundation Trust, Hills Road, Cambridge, CB2 0QQ, United Kingdom

Email: [ab2031@cam.ac.uk](mailto:ab2031@cam.ac.uk)

Twitter: @HPBCambridge

Tel: +44 1223 256 151

**ORCID ID :** 0000-0002-9172-3938

**Supplementary Materials - Index**

| **Supplementary Methods** |  | | |
| --- | --- | --- | --- |
| Statistical analysis | *page 4* | | |
| **Supplementary Figures and Tables** |  | | |
| Figure S1 – Distribution of countries participating in the OMEGA study (marked by red squares) superimposed on estimated global age standardized incidence rates (ASR) of gallbladder cancer per 100,000 individuals. Adapted with permission from the International Agency for Research on Cancer’s GLOBOCAN database | | *page 5* | |
| Figure S2 – Logistic regression of A) morbidity and mortality and B) risk of node positive disease associated with extended lymphadenectomy | | | *page 6* |
| Figure S3 – Multivariable analysis of the effects of a) extent of nodal dissection and b) total lymph nodes excised (TLNE) on recurrence-free survival | | *page 7* | |
| Figure S4 – Analysis of recurrence-free survival by nodal staging model, a) 8^th^ AJCC classification, b) 7^th^ AJCC classification, c) positive lymph node ratio of 0.1 | | | *page 8* |
| Figure S5 - Multivariable regression of recurrence-free survival by a) sites of positive nodes, b)7^th^ AJCC classification, c) positive lymph node ratio of 0.1, d) positive lymph node ratio as a continuous variable  Figure S6 – Modified logistic regression analysis of node positive status for risk prediction score  Figure S7 - Calibration curves for the train dataset (a), and the test dataset (b) | | | *page 9*  *page 10*  *page 11* |
|  |  | | |

**Supplementary Methods**

**Data collection:**

GBC incidence was determined using Globocan 2020 data, using the top quartile (>1·1 GBC cases per 100,000 population) to define “high” incidence^2^.

Statistical analysis:

Data on TLNE were analysed using Poisson and Negative Binomial regression models. The models were estimated under the Bayesian paradigm via the No-U-Turn-Sampler, a variant of Hamiltonian Monte Carlo. The best model (Negative Binomial regression in this case) was selected using the widely applicable information criterion (WAIC) and the Leave-one-out cross-validation (LOO-CV) criterion. As a sensitivity analysis, we also examined zero-inflation versions of these models. Binary data were analysed using a number of models: logistic regression, recursive partitioning classification tree and random forest. The suitable covariates in logistic regression were determined using forward-backward stepwise regression via the Akaike information criterion (AIC) selection method.  Survival data were analysed using the Cox model and Kaplan Meier plots. Separate multivariable analyses excluding patients with Nx status were employed to assess the association of nodal parameters such as positive node ratio and AJCC nodal classification on RFS due to collinearity between those variables. Recursive partitioning classification trees were also used to select the most suitable cut-off value on analysis of PLNN and PLNR. The proportionality assumption of the Cox model was tested using deviance and scaled Schoenfield residuals. The predictive ability and the goodness-of-fit of the prognostic models (PLNR and AJCC classification) were compared using AIC and C-statistic. The C-statistic confidence intervals (CIs) were derived using point estimate ± 1.96*standard error and a Wald-type test against poor predictive ability was performed by inspecting for C-statistics which contained 0.5.

For the development of the risk prediction model for node involvement, we split the dataset to 80% and 20% train and test sets and performed 10-fold cross-validation repeated 10 times on the train dataset to select our hyperparameters for the tree and random forest on the train dataset. We validated the predictive discrimination of the risk score on the separate test dataset. Models were evaluated by cross-validation on multiple validation sets to provide a more realistic estimate of the model’s generalizability and ability to perform well on new, unseen data.  The final risk score was derived by (i) multiplying the coefficients of the logistic regression model by 3, (ii) rounding to the nearest integer, (iii) adding the transformed coefficient and (iv) allocating patients into brackets based on their probability of being node positive.

**Supplementary Figures and Tables**


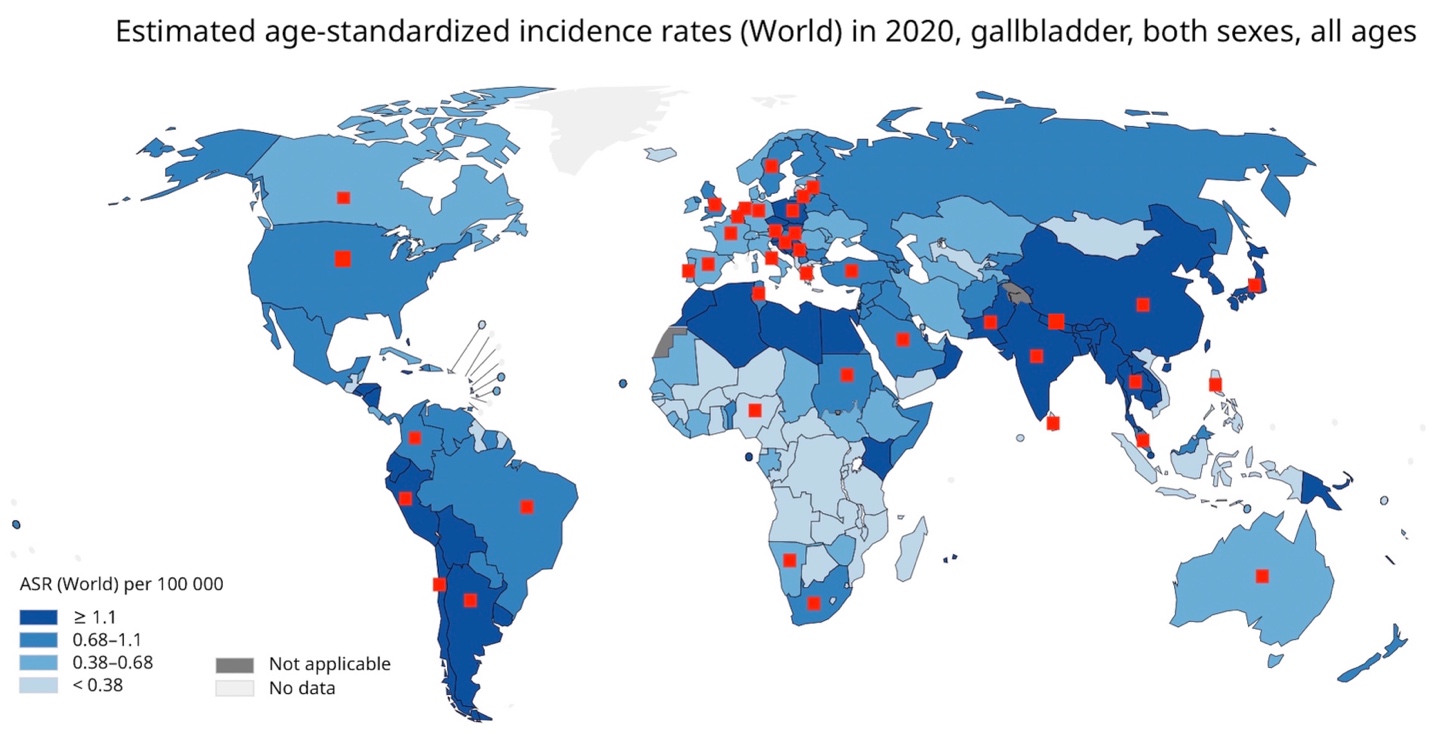


Figure S1: Distribution of countries participating in the OMEGA study (marked by red squares) superimposed on estimated global age standardized incidence rates (ASR) of gallbladder cancer per 100,000 individuals. Adapted with permission from the International Agency for Research on Cancer’s GLOBOCAN database (reference 2).

Figure S2 – Logistic regression of A) morbidity and mortality and B) risk of node positive disease associated with extended lymphadenectomy. EBDR – extrahepatic bile duct resection, LVPI – lymphovascular and perineural invasion

Figure S3 – Multivariable analysis of the effects of a) extent of nodal dissection and

b) total lymph nodes excised (TLNE) on recurrence-free survival. EBDR- extrahepatic bile duct resection

Figure S4 – Analysis of recurrence-free survival by nodal staging model, a) 8^th^ AJCC classification, b) 7^th^ AJCC classification, c) positive lymph node ratio [PLNR] with cut-off of 0.1

Figure S5 - Multivariable regression of recurrence-free survival by a) sites of positive lymph nodes, b)7^th^ AJCC classification, c) positive lymph node ratio of 0.1, d) positive lymph node ratio as a continuous variable. EBDR – extrahepatic bile duct resection

Figure S6 – Modified logistic regression analysis of risk of node positive status for risk prediction score. LVPI – lymphovascular and perineural invasion


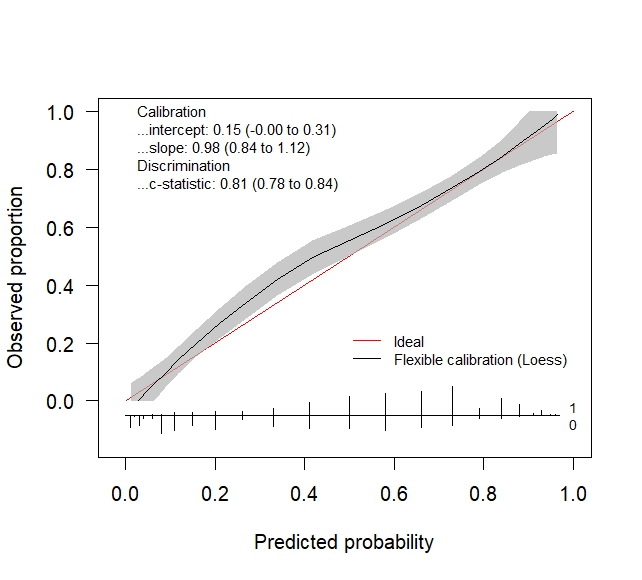


A


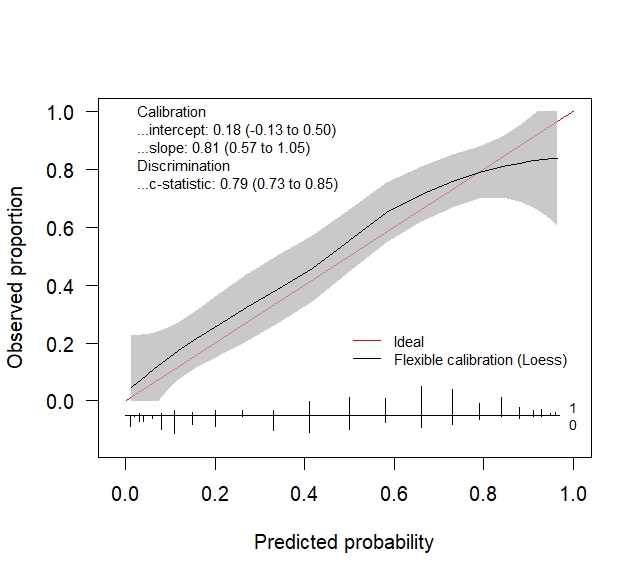


B

Figure S7: Calibration curves for the train dataset (a), and the test dataset (b).
